# Supplementary material for: Technology availability, sector policies and behavioral change are complementary strategies for achieving net-zero emissions
Source: Nat Commun. 2024 Sep 30;15:8440. doi: 10.1038/s41467-024-52424-0 (PMC11445258; doi:10.1038/s41467-024-52424-0)
Supplement: Supplementary file 1 — Supplementary Information [file 41467_2024_52424_MOESM1_ESM.pdf]

Supplementary Information

Technology availability, sector policies and behavioral change are complementary strategies for achieving net-zero emissions

Jessica Strefler*[1], Leon Merfort[1], Nico Bauer[1], Miodrag Stevanović[1], Dennis Tänzler[2], Florian Humpenöder[1], David Klein[1], Gunnar Luderer[1,3], Michaja Pehl[1], Robert C. Pietzcker[1], Alexander Popp[1,4], Renato Rodrigues[1], Marianna Rottoli[1], Elmar Kriegler[1,5]

[1]*Potsdam Institute for Climate Impact Research (PIK), Member of the Leibniz Association, PO Box 601203, 14412 Potsdam, Germany*
[2]*adelphi consult GmbH, Alt-Moabit 18, 10559 Berlin, Germany*
[3]*Technische Universität Berlin, Berlin, Germany*
[4]*Faculty of Organic Agricultural Sciences, University of Kassel, Germany*
[5]*Universität Potsdam, Potsdam, Germany*

* Corresponding author, eMail strefler@pik-potsdam.de, phone +49 331 288 2475

Contents:

Supplementary Note 1: Description of stakeholder engagement
Supplementary Note 2: Description of transformation measures
Supplementary Note 3: GHG-neutral scenarios
Supplementary Figures
Supplementary References

Supplementary Note 1: Description of stakeholder engagement

When developing the transformation framework for deep decarbonisation, a particular challenge is to understand the motivations and needs of various stakeholders and to take them into account as far as possible. This requirement was actively addressed as part of the project and contributed significantly to the concretisation of the scenario narratives.

To this end, an iterative co-creation process was designed and implemented over three years to discuss the scenario assumptions and interim results with selected representatives from business (Federation of German Industries, BDI, German Association of Energy and Water Industries, BDEW), civil society including consumer protection (Vzbv, WWF, Misereor, Germanwatch) and administration (Federal Environment Agency). It was possible to involve largely the same representatives of the institutions over the three years. Together, these stakeholders from politics, business and civil society have contributed significantly to the development and review of the specific series of scenario narratives through three joint integrated and additional separate rounds of commentary.

In a first workshop, researchers introduced their models and the process of scenario design, and showed first results of transformation scenarios. Based on this, the stakeholders discussed visions for the year 2050 as well as levers and hurdles in the areas technology & innovation, economy & consumption, political framework conditions, distribution issues and opportunities for sector coupling along the five sectors power, transport, industry, buildings, and agriculture. These inputs were used to develop seven different scenario narratives based on the transformation measures the stakeholders suggested in the five sectors "electricity", "transport", "buildings", "industry" and "agriculture/land use" along the three dimensions technology and innovation, political coordination, and behavioural change (see Supplementary Table 1).

In this second workshop, the stakeholders discussed and refined the scenario narratives, resulting in a reduction to six scenarios. In addition, they developed "personas" as a core methodological element. As figures alone are often not enough to make the scenarios tangible, these were developed together with the stakeholders. "Personas" are fictitious representatives of a target group and have goals and needs. The aim here was not to depict an average of the population, but rather specific individuals who illustrate patterns in user behaviour. In the course of an interactive discussion of the scenarios and interim results, this approach of co-production of scenario narratives was chosen and different "personas" were characterised as either supporters or opponents of such a transformation against the background of the transformations set out in the scenarios. This broader perspective can help to take greater account of existing reservations and motives of the population in the political design of the transformation paths. The year 2030 was defined as the time frame, from which a retrospective forecast was made (REgnose), i.e. a look back from the future that shows the developments from 2030 that could have characterised the path up to 2030. The results of the six scenarios were presented in a final stakeholder workshop and discussed again, resulting in the final set of five scenarios.

Supplementary Note 2: Description of transformation measures

Supplementary Table 1 shows the transformation measures for the EU+UK used in the scenarios. The rest of the world has no restrictions on technologies, no specific sector policies, and no behavioral change, i.e., the emissions reduction there is purely CO_2eq-price driven.

Technology & Innovation

	Focus GHG reduction	Focus social acceptance
**CCS**	Maximum 750 Mt CO_2/yr, DACCS available	Maximum 150 Mt CO_2/yr, no DACCS
**Bioenergy**	No exports; beyond this no further constraints	Only residues + maximum 2 EJ/yr, no exports or imports
**Nuclear energy**	No constraints	No new investments past 2020
**Wind energy**	No constraints	Lack of social acceptance leads to higher expansion costs

Political coordination

	Sector oriented	Market oriented
**Industry**	• Promotion of direct electrification through subsidies of the electricity price.<br>• Promotion of indirect electrification through subsidies of hydrogen production	Only CO_2eq-price driven
**Buildings**	Ban of coal, oil, and gas heating by 2050	Only CO_2eq-price driven
**Transport**	• Targeted promotion of electromobility leads in particular to significantly faster introduction of electric cars<br>• Ban on the sale of new internal combustion vehicles from 2030 onwards	Only CO_2eq-price driven
**Land**	• Precision Agriculture: Increase of nitrogen & irrigation water use efficiency.<br>• Price limit for CH_4 and N_2O emissions	Only CO_2eq-price driven

Behavioral change

	Value oriented	Price oriented
**Buildings**	Sustainable lifestyle leads to lower final energy demand[1]	Only CO_2eq-price driven[1]
**Transport**	• Modal shift towards public and active transport modes[2,3]<br>• Reduced number of person-kilometers and reduced consumption lead to lower final energy demand[2,3]	Only CO_2eq-price driven[2,3]
**Land**	• EAT-Lancet diet[4]<br>• Less food waste[5,6]	Continuation of current trends for food demand and composition

Supplementary Table 1: List of all transformation measures.

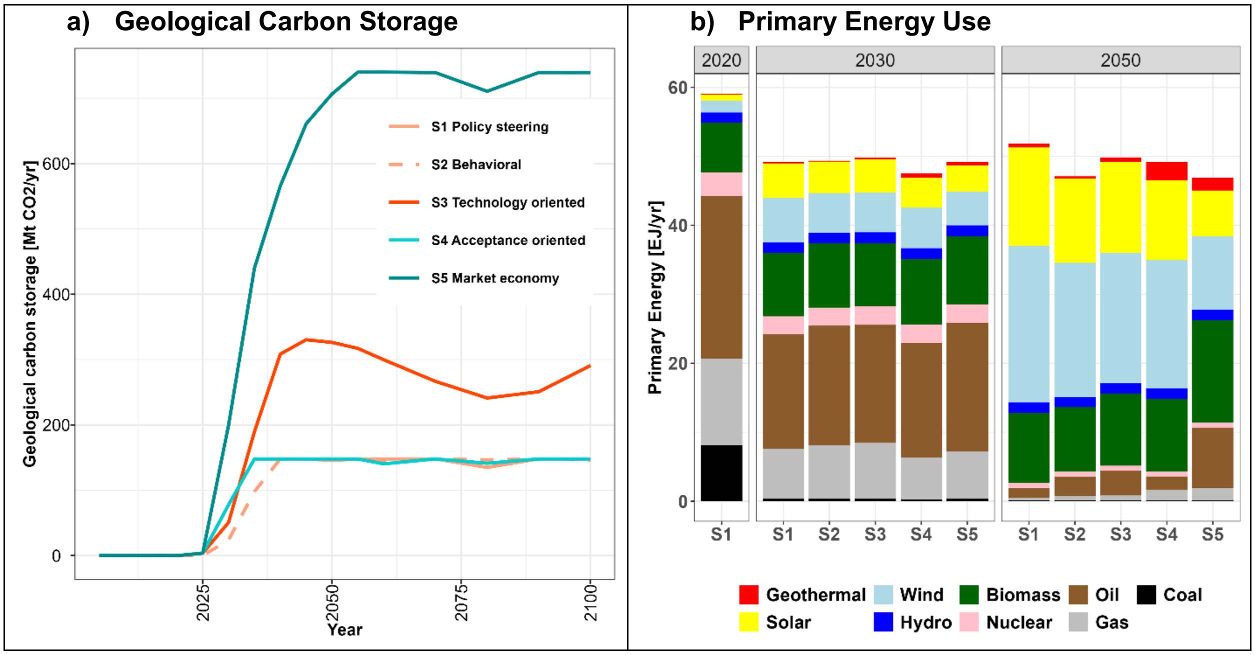

Supplementary Figure 1: Impacts of the technology and innovation dimension in the European Union. a) geological carbon storage and b) primary energy use in 2020, 2030 and 2050.

Supplementary Figure 1 shows impacts of the technology dimension. The limit on CCS in scenarios with focus social acceptance (S1, S2, S4) is clearly binding, while the limit on nuclear expansion has no impact due to the high costs of nuclear. For wind power, there is no hard limit but an increase in costs, such that only a relative reduction is visible for S3 vs. S1 and S5 vs. S4.

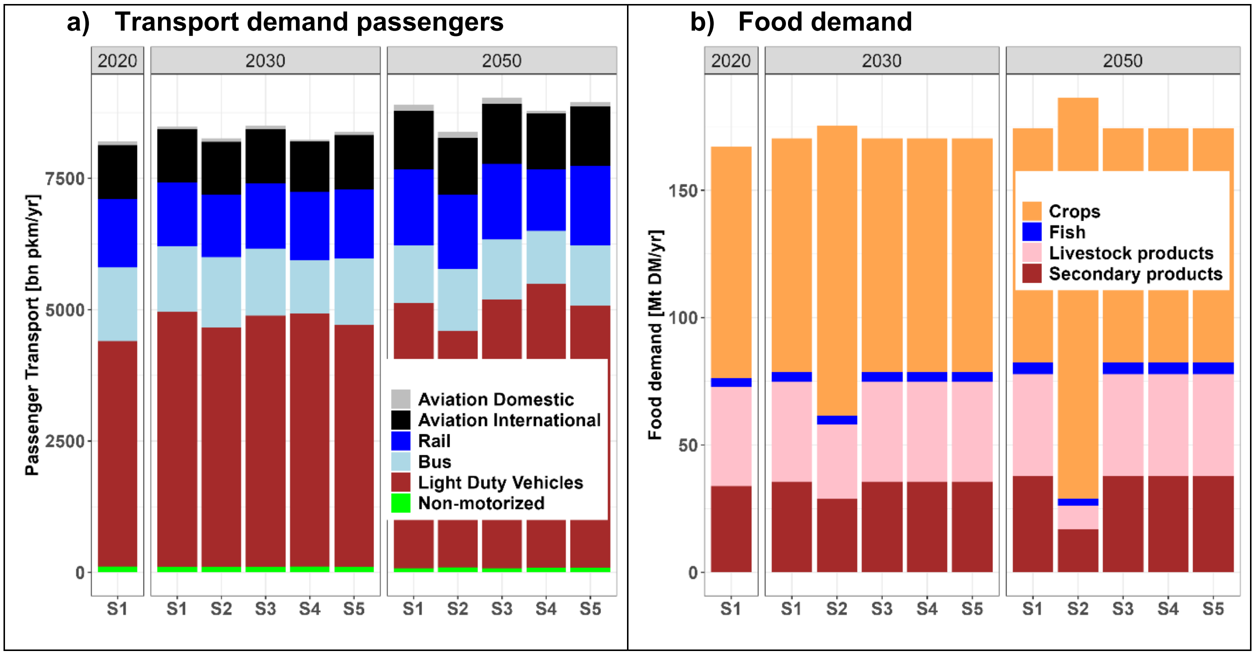

Supplementary Figure 2: Impacts of the behavioural change dimension on mobility and food demand in the European Union in 2020, 2030, and 2050. Demand for a) passenger transport kilometers and b) food.

The main impacts of the measures in the political coordination dimensions are shown in Figure 3 in the main text. The additional sector policies in scenarios S1-S3 lead to a phase out of combustion engine vehicles (Figure 3a) and fossil fuels in the building sector (Figure 3b) by 2050 and to more electricity use in all sectors (Figure 3c).

The behavioural changes in scenario S2 on the buildings sector show in Figure 3b in the main text, where S2 has a significantly lower final energy demand than the corresponding scenario S1. Figure 3a shows that the shift to active transport modes and the reduced number of person-kilometers leads to a reduction of the number of vehicles. The modal split of person-kilometers is shown in Supplementary Figure 2a. The dietary changes are detailed in Supplementary Figure 2b. The behavioural changes in scenario S2 lead to a significant reduction in secondary products (like sugar and alcoholic beverages) and livestock products, which are replaced by crops as rice or fruits, vegetables and nuts, according to the EAT-Lancet dietary recommendations.

Supplementary Note 3: GHG-neutral scenarios

The two scenarios with limited technology availability and price-oriented behavior S1 and S4 cannot achieve GHG neutrality in 2050 because there is not enough CDR available to compensate for the remaining residual emissions, especially of CH_4 and N_2O (Supplementary Figure 3). Thus, to achieve GHG neutrality, at least one of the two approaches, behavioral change or full technology availability leading to more CDR, must be pursued. In the following we will compare the remaining three scenarios S2, S3, S5 with a scenario variant that achieves GHG-neutrality in 2050 S2*, S3*, S5*.

The emission trajectories for all GHG-neutral scenarios (*S2, *S3, *S5) are again similar (Supplementary Figure 4a). In 2050, in the scenario with limited availability of CDR *S2, residual emissions need to be further reduced, especially in the transport sector (Supplementary Figure 3). This is accompanied by a significant increase in the CO_2 price by more than 70% from 110 €/tCO_2 to 190 €/tCO_2 in 2030 (Supplementary Figure 4b). In S3, sector policies had already ensured a reduction in residual emissions, so there is less need for CDR. In the GHG-neutral scenario *S3, CDR, mainly BECCS, is now increased, residual emissions change little, and the CO_2 price increases from 125 €/tCO_2 to 160 €/tCO_2 in 2030. In contrast, S5 had allowed more residual emissions, which are offset by high levels of CDR. Since no more CDR is available, also in *S5 residual emissions have to be reduced, especially in the industrial and transport sectors, to achieve GHG neutrality. The CO_2 price increases from 194 €/tCO_2 to 229 €/tCO_2 in 2030.

All scenarios have in common that they use significantly more biomass and therefore also more area for biomass production, either to expand BECCS, or to reduce residual emissions in industry and transport through biofuels (Supplementary Figure 5).

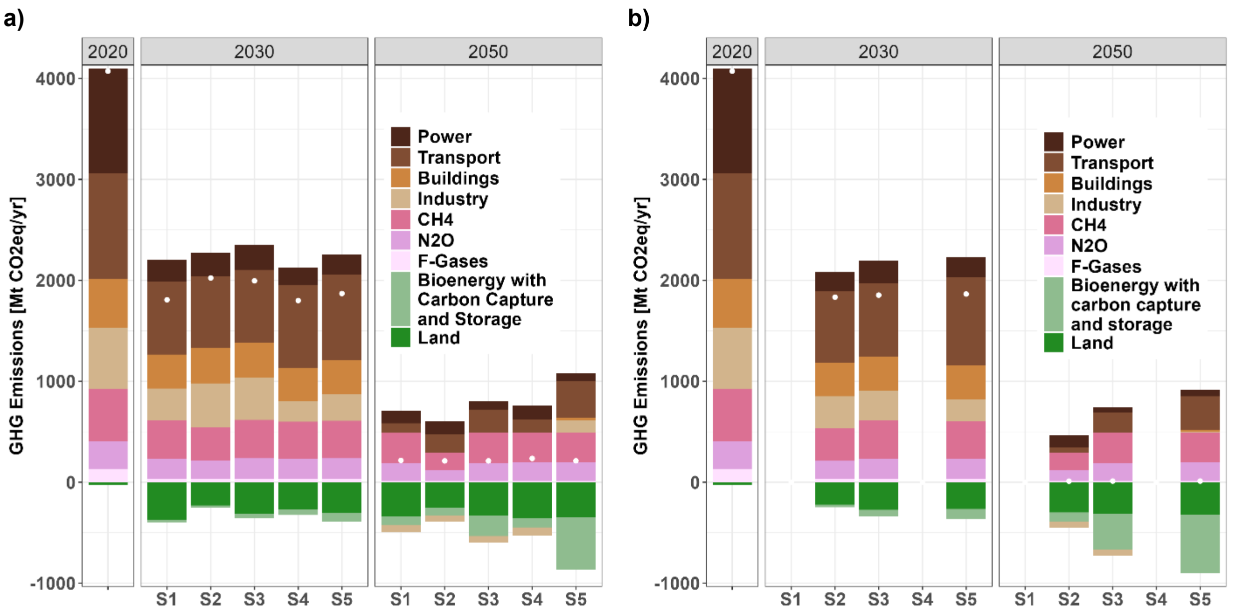

Supplementary Figure 3: Greenhouse gas emissions by gas in the European Union in 2020, 2030, and 2050, with CO_2 emissions also disaggregated by sector. a) all default scenarios and b) the three scenarios achieving greenhouse gas neutrality in 2050.

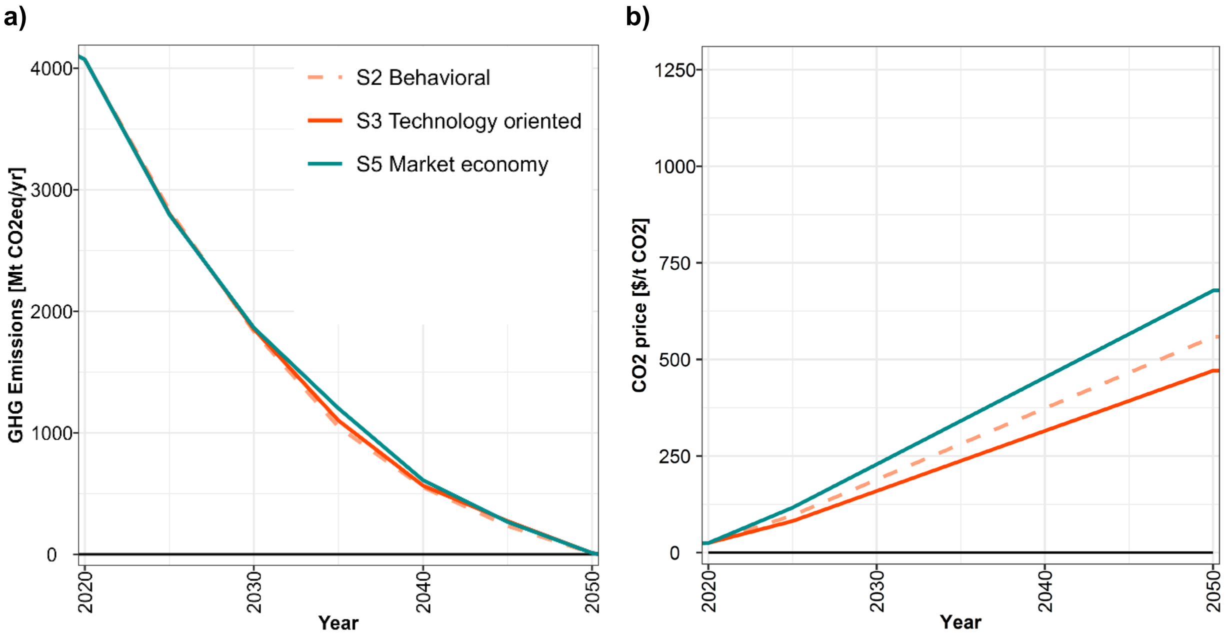

Supplementary Figure 4: Key characteristics of the three scenarios achieving greenhouse gas neutrality in the European Union in 2050. a) Annual greenhouse gas emissions and b) carbon prices.

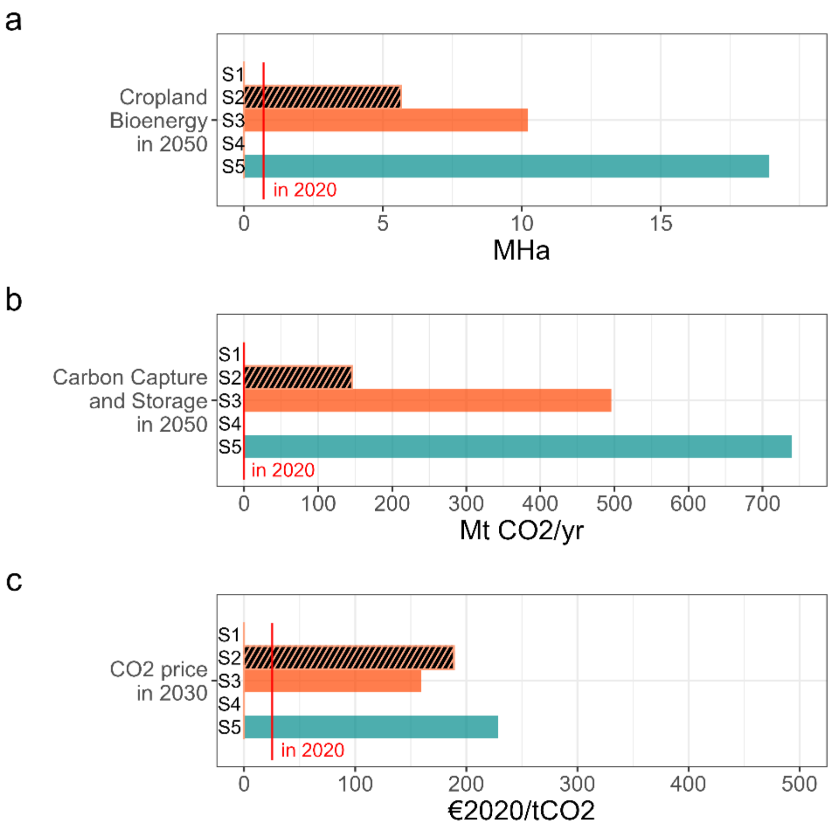

Supplementary Figure 5: Key trade-off for the technology and innovation dimension in the European Union. A) Cropland for bioenergy and b) carbon capture and storage (values shown for 2050) increase, while c) carbon price (values shown for 2030) decreases. We also indicate the respective values in 2020.

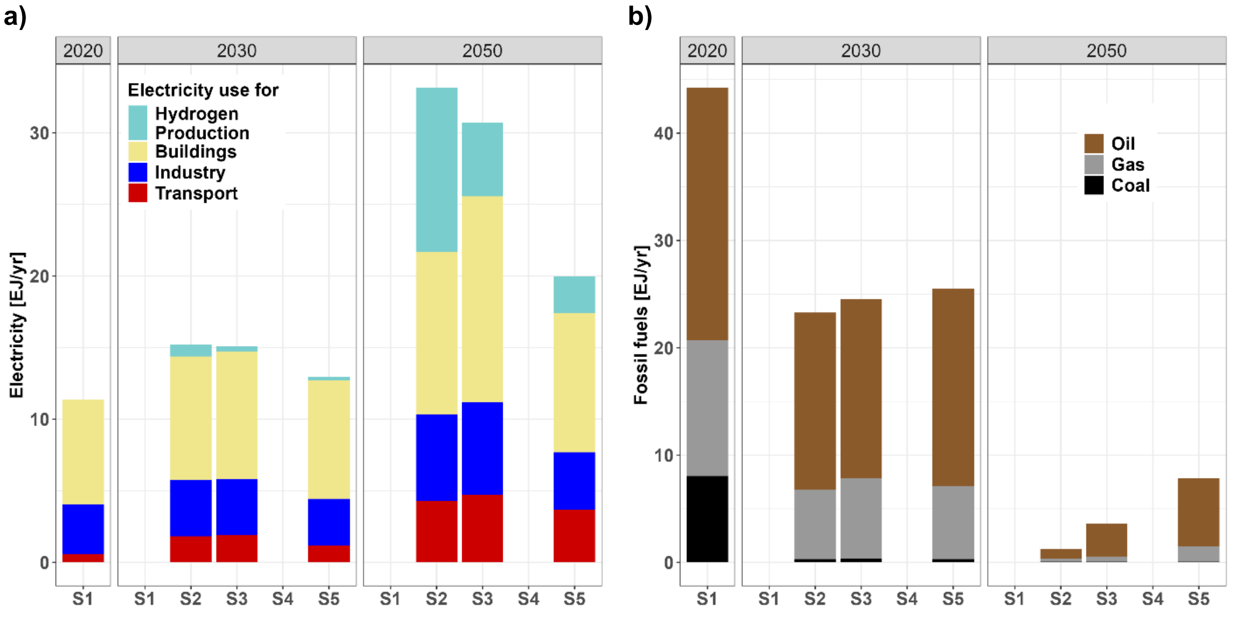

Supplementary Figure 6: Impacts of sector policies on the energy system the European Union. a) Electricity demand by sector and b) fossil fuel use by source.

Supplementary Figures

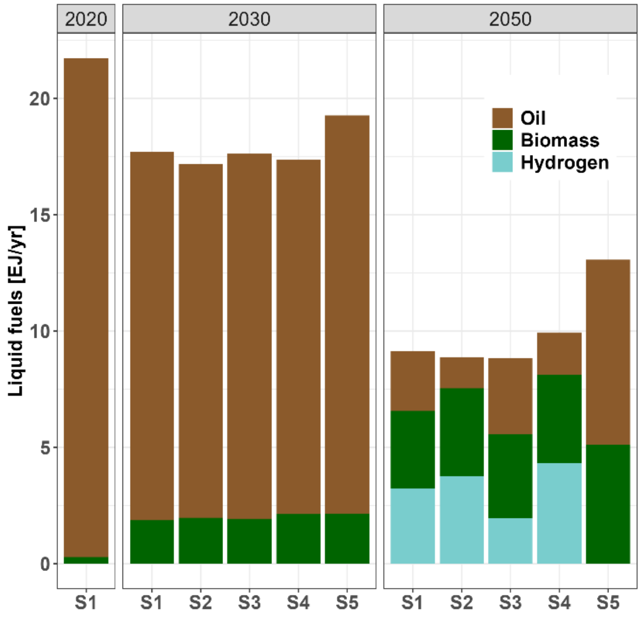

Supplementary Figure 7: Liquid fuel use the European Union in 2020, 2030, and 2050 by primary energy source. The enhanced direct electrification in scenarios S1-S3 reduces the demand for liquid fuels. In addition, oil is increasingly replaced by biomass and hydrogen, reducing oil use by 63-94% by 2050 as compared to 2020.

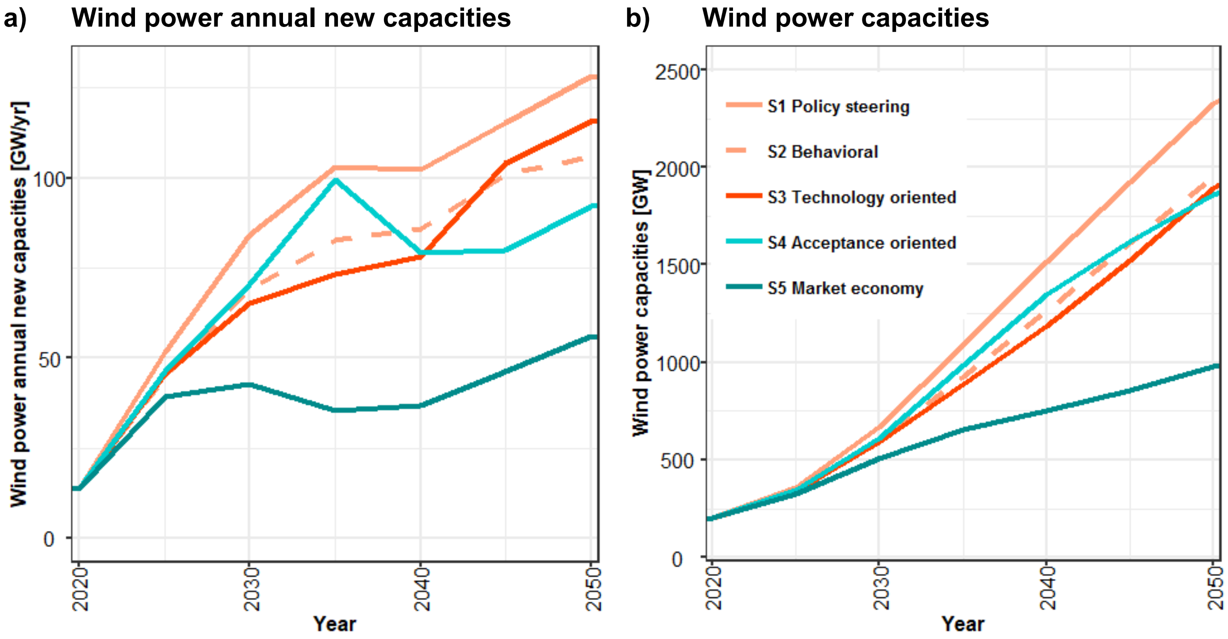

Supplementary Figure 8: Wind power capacities in the European Union. a) annual new capacities and b) total capacities. The enhanced direct electrification in scenarios S1-S3 and indirect electrification in scenarios S1-S4 requires a faster expansion of renewable energy. Already in 2030, wind energy annual new capacities are about a factor of 4 higher in S1-S4 than in 2020.

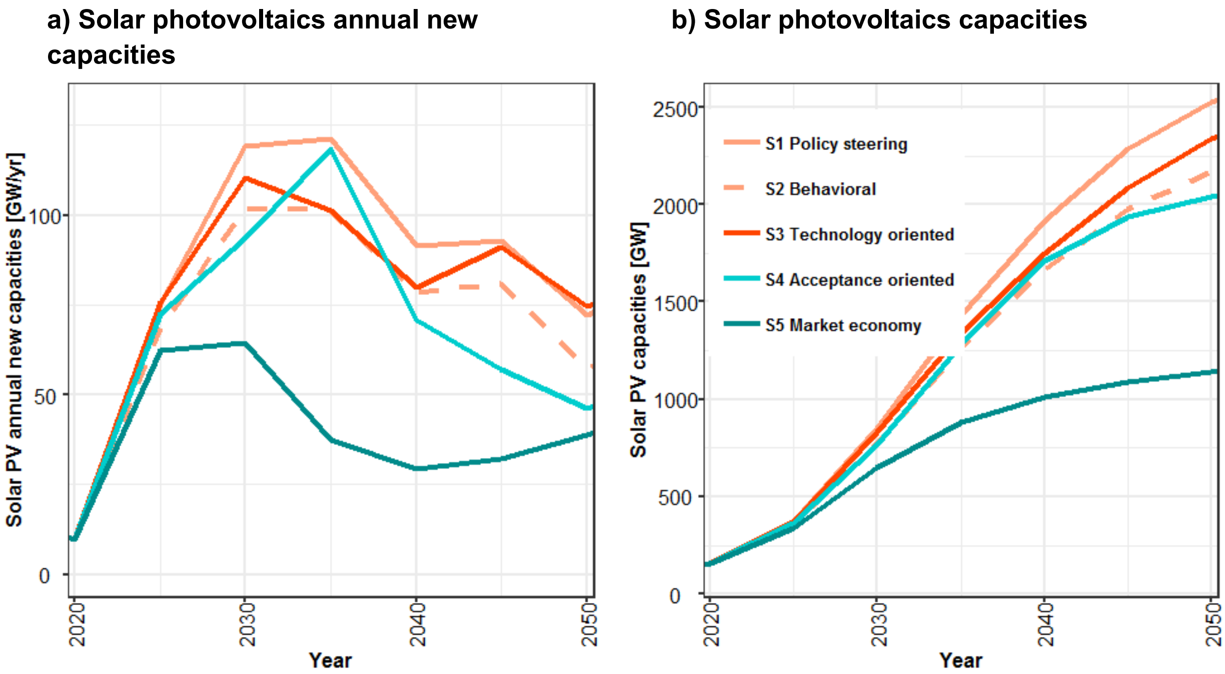

Supplementary Figure 9: Solar photovoltaic capacities the European Union. a) annual new capacities and b) total capacities. The enhanced direct electrification in scenarios S1-S3 and indirect electrification in scenarios S1-S4 requires a faster expansion of renewable energy. Already in 2030, solar photovoltaics annual new capacities are about a factor of 10-11 higher in S1-S4 than in 2020.

Supplementary References

1. Levesque, A. *et al.* How much energy will buildings consume in 2100? A global perspective within a scenario framework. *Energy* **148**, 514–527 (2018).
2. Rottoli, M., Dirnaichner, A., Pietzcker, R., Schreyer, F. & Luderer, G. Alternative electrification pathways for light-duty vehicles in the European transport sector. *Transp. Res. Part Transp. Environ.* **99**, 103005 (2021).
3. Dirnaichner, A. *et al.* Life-cycle impacts from different decarbonization pathways for the European car fleet. *Environ. Res. Lett.* **17**, 044009 (2022).
4. Willett, W. *et al.* Food in the Anthropocene: the EAT–Lancet Commission on healthy diets from sustainable food systems. *The Lancet* **393**, 447–492 (2019).
5. Humpenöder, F. *et al.* Projected environmental benefits of replacing beef with microbial protein. *Nature* **605**, 90–96 (2022).
6. Soergel, B. *et al.* A sustainable development pathway for climate action within the UN 2030 Agenda. *Nat. Clim. Change* **11**, 656–664 (2021).
